# LOX-1: A potential driver of cardiovascular risk in SLE patients

**DOI:** 10.1371/journal.pone.0229184

**Published:** 2020-03-17

**Authors:** Divya Sagar, Ranjitha Gaddipati, Emily L. Ongstad, Nicholas Bhagroo, Ling-Ling An, Jingya Wang, Mehdi Belkhodja, Saifur Rahman, Zerai Manna, Michael A. Davis, Sarfaraz Hasni, Richard Siegel, Miguel Sanjuan, Joseph Grimsby, Roland Kolbeck, Sotirios Karathanasis, Gary P. Sims, Ruchi Gupta

**Affiliations:** 1 Respiratory, Inflammation and Autoimmune, Biopharmaceuticals R&D, AstraZeneca, Gaithersburg, Maryland, United States of America; 2 Cardiovascular, Renal and Metabolism, Biopharmaceuticals R&D, AstraZeneca, Gaithersburg, Maryland, United States of America; 3 National Institute of Arthritis and Musculoskeletal and Skin Diseases, National Institute of Health, Bethesda, Maryland, United States of America; Centro Cardiologico Monzino, ITALY

## Abstract

Traditional cardiovascular disease (CVD) risk factors, such as hypertension, dyslipidemia and diabetes do not explain the increased CVD burden in systemic lupus erythematosus (SLE). The oxidized-LDL receptor, LOX-1, is an inflammation-induced receptor implicated in atherosclerotic plaque formation in acute coronary syndrome, and here we evaluated its role in SLE-associated CVD. SLE patients have increased sLOX-1 levels which were associated with elevated proinflammatory HDL, oxLDL and hsCRP. Interestingly, increased sLOX-1 levels were associated with patients with early disease onset, low disease activity, increased IL-8, and normal complement and hematological measures. LOX-1 was increased on patient-derived monocytes and low-density granulocytes, and activation with oxLDL and immune-complexes increased membrane LOX-1, TACE activity, sLOX-1 release, proinflammatory cytokine production by monocytes, and triggered the formation of neutrophil extracellular traps which can promote vascular injury. In conclusion, perturbations in the lipid content in SLE patients’ blood activate LOX-1 and promote inflammatory responses. Increased sLOX-1 levels may be an indicator of high CVD risk, and blockade of LOX-1 may provide a therapeutic opportunity for ameliorating atherosclerosis in SLE patients.

## Introduction

Systemic lupus erythematosus (SLE) is a chronic, autoimmune disease that leads to multi-organ damage and degradation of connective tissue primarily through inflammation. Although cardiovascular damage related fatalities remain the leading cause of all mortality worldwide, SLE associated inflammatory risk factors independently contribute to a rapid acceleration of premature atherosclerosis [1, 2]. Independent clinical studies show strong evidence that patients with SLE have at least two- to three-fold higher risk of heart disease and stroke, compared to individuals without SLE [3]. Adjusting for traditional cardiovascular risk factors such as age, sex, BMI, cholesterol, systolic blood pressure and statin usage based on the Framingham Heart Study still leaves a significantly high number of SLE patients with unfavorable cardiovascular outcomes [4].

Therefore, understanding the risk factors that make SLE patients more prone to CVD is an important area of research. Further understanding which SLE patients are more susceptible to CVD can assist in monitoring and treating these patients for CVD early on.

SLE patients frequently show elevated levels of triglycerides, total cholesterol, very low density lipoprotein cholesterol (VLDL-C) and low levels of high density lipoprotein cholesterol (HDL-C) [5]. Along with the reduced HDL levels in SLE [6–8], HDL function is also highly compromised. Proinflammatory HDL (piHDL), defined by its pro-oxidant nature, is an important measure for lipid dysregulation in SLE associated cardiovascular disease. In a `prospective study comprised of 276 women with SLE, McMahon et al. deduced that piHDL was the most significantly associated CVD risk factor in SLE patients with carotid plaques [9]. piHDL was present in 86.7% of SLE patients with atherosclerotic coronary plaques and positively correlated with plaque intima thickness (IMT) [10]. Consistent with this observation, elevated oxidized LDL (oxLDL) has been reported in SLE patients with verified subclinical atherosclerosis [11].

An additional measure of HDL functionality, namely cholesterol efflux capacity (CEC) or the ability of HDL to remove cholesterol from macrophages in-vitro, is impaired as a result of HDL chlorination in human atherosclerotic lesions [12, 13]. Chlorination, an outcome of myeloperoxidase (MPO)-catalyzed oxidation [14], has been further shown to be enhanced by MPO released by neutrophil extracellular trap (NET) formation from SLE low density granulocytes (LDGs), a specialized subset of neutrophils [15].

oxLDL, dysfunctional HDL and hsCRP are atherogenic ligands that bind to the scavenger receptor LOX-1, expressed on endothelial cells, monocytes, macrophages, smooth muscle cells and platelets [16]. LOX-1 deficiency protects LDLR^-/-^ atherosclerosis prone mice from development of plaques [17]. LOX-1 overexpression worsens atherosclerosis in murine models of atherogenesis [18]. *In vitro* studies on human LOX-1 receptor signaling show that ligand binding to LOX-1 increases ROS production and activates arginase [19] both of which inhibit endothelial NO production making endothelium stiff and dysfunctional [20]. In addition, LOX-1 activation induces apoptosis [21], macrophage foam cell formation [22] and vascular smooth muscle cell proliferation [23] primarily through a feed-forward system stimulated by oxLDL, a major atherogenic ligand. Recently, inflammatory HDL from SLE patients was shown to reduce the nuclear co-localization of ATF3, an anti-inflammatory transcription factor, through the LOX-1 receptor, making primary human monocyte-derived macrophages prone to inflammatory cytokine secretion [24]. In human disease, elevated levels of cleaved soluble LOX-1 (sLOX-1) have been reported in patients with acute coronary syndrome [25, 26], systolic heart failure [27], ischemic stroke [28], and psoriasis [29].

Cell bound LOX-1 receptor can be cleaved by ADAM10 and ADAM17 (also known as TACE, TNF-α converting enzyme) in the neck region of extracellular domain to release sLOX-1 [30, 31]. Importantly, high levels of sLOX-1 associated with carotid plaque inflammation and future risk of ischemic stroke in a study involving analysis of sLOX-1, plaque LOX-1, lipid profiles and plaque presence in 4703 subjects from the Malmö Diet and Cancer (MDC) cohort during a mean follow‐up of 16.5 years [32]. Recently, we showed that sLOX-1 levels track with the burden of non-calcified coronary plaques in psoriasis patients [29]. We hypothesize that sLOX-1 may serve as a biomarker of enhanced LOX-1 expression in SLE patients with subclinical atherosclerosis. In our study, we have investigated the role of LOX-1 in SLE and evaluated the potential of sLOX-1 as a biomarker for assessing cardiovascular risk, beyond the traditional cardiovascular risk predictors. In 273 SLE donors and 72 healthy controls, we report an elevation of sLOX-1 in 35% patients with SLE. SLE patients with high sLOX-1 levels had elevated hsCRP, triglycerides, oxLDL, piHDL and impaired HDL CEC compared to the low sLOX-1 group. We also show that modified lipids from SLE patients function as LOX-1 ligands contributing to inflammatory immune activation through proinflammatory cytokine secretion from monocytes and enhanced NET formation by LDGs. These data establish a mechanism of action whereby sLOX-1 levels can be predictive of vascular inflammation, highlighting the potential for LOX-1 receptor blockade as a target for preventing binding of various atherogenic ligands and ameliorating cardiovascular damage in SLE.

## Results

### sLOX-1 is elevated in SLE patients and is higher in patients up to 40 years of age

**Fig 1:**
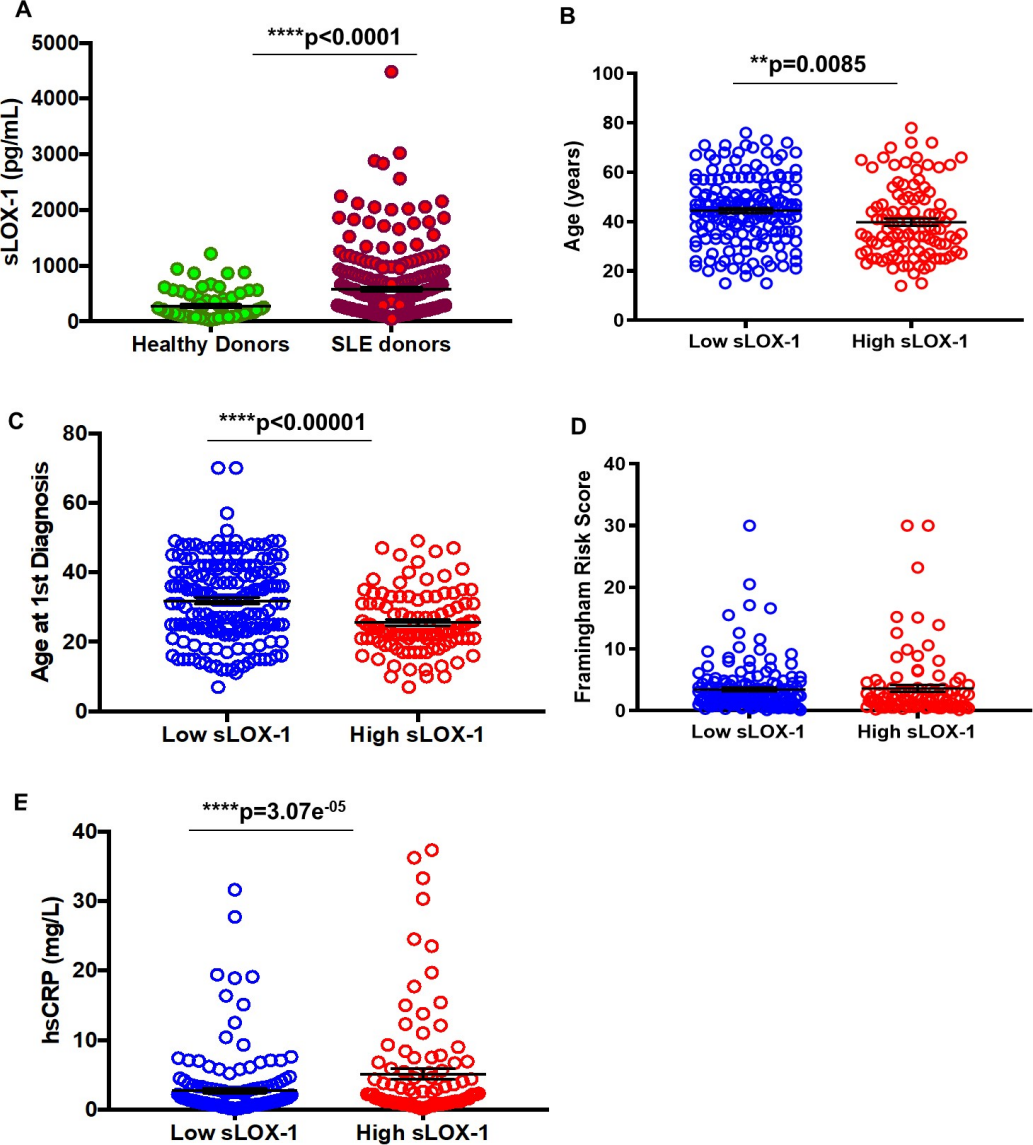
Measurement of sLOX-1 levels in healthy versus SLE donors and relationships between sLOX-1 and age, Framingham risk scores (FRS) and hsCRP. (A) Serum from healthy (n = 72; sLOX-1 pg/mL mean = 274.9 ± 30.34 SEM) and SLE (n = 273; sLOX-1 pg/mL mean = 580.9 ± 36.1) individuals were measured for levels of sLOX-1 using an MSD-based ELISA assay. Bars represent mean ± standard error (SEM). (B) Age of patients with low sLOX-1 levels <532 pg/mL (44.53 ± 1.035 years, n = 171) and high sLOX-1 levels >532 pg/mL (39.82 ± 1.442 years, n = 102) were analyzed. (C) Age of first SLE diagnosis in patients with low sLOX-1 (31.83 ± 0.9143, n = 170) versus high sLOX-1 (25.55 ± 0.8747, n = 103) levels. (D) 10-year FRS were calculated based on patients’ sex, age, systolic blood pressure (SBP), use of antihypertensive treatment, smoking and diabetes, cholesterol and LDL cholesterol levels. (E) Clinical hsCRP (mg/dL) measurements were used to analyze levels between low and high sLOX-1 patients. * p<0.05, ** p<0.01, ***p<0.001 and ****p<0.0001.

sLOX-1 was measured in age- and sex- matched healthy donors (n = 72) and SLE patients (n = 273) (S1 Table) using an MSD-based ELISA assay. We found serum sLOX-1 to be significantly increased in a subset of SLE patients compared to healthy donors (Fig 1A), with the mean for SLE at 580.9 ± 36.1 pg/mL compared 274.9 ± 30.34 pg/mL in healthy donors (p<0.0001). A cut-off at 532 pg/mL sLOX-1 (mean plus one standard deviation of healthy donors) was used to define high and low sLOX-1 levels. Patients in the high sLOX-1 group were younger (39.82 ± 1.442 years) compared to the low sLOX-1 group (44.53 ± 1.035 years; p<0.0014) (Fig 1B and Table 1). There was no difference in disease duration between the two groups (Table 1), indicating that high sLOX-1 is associated with an earlier onset of SLE. Age of SLE diagnosis was indeed significantly lower (25.55 ± 0.87 years) in the high sLOX-1 group versus low sLOX-1 group (31.83 ± 0.9143; p<0.0001) (Fig 1C and Table 1).

**Table 1:** Association between sLOX-1 and disease duration, serological and clinical features.

Observation	Low LOX-1 (n)	Low	High LOX-1 (n)	High	P value (Group comparison)	Spearman Correlation (r_s_)	P value (r_s_)
		LOX-1 (Median (IQR)/%)		LOX-1 (Median (IQR)/%)			
**Age (yrs)**	170	45 (36–53)	103	37(28–49)	**8.50E-03**	-0.17	**5.80E-03**
Disease Duration (yrs)	170	10(5–19)	103	11(5–22)	0.2	0.03	0.61
**Age at 1**^**st**^ **Diagnosis**	170	32 (23–41)	103	23(21–31)	**<0.0001**	-0.26	**<0.0001**
**C3 (mg/dL)**	170	94.8 (80.9–108.7)	103	109 (91.8–131)	**4.80E-07**	0.36	**1.30E-09**
**C4 (mg/dL)**	169	18.4 (12.6–23.6)	103	22 (15.4–25.2)	**4.98E-03**	0.22	**2.09E-04**
**Platelets (# per μl WB)**	170	224.5 (182–270)	103	243 (202–300)	**0.02**	0.19	**1.32E-03**
ESR (mm/hr)	168	19 (11.5–38)	102	21 (12.5–34)	0.74	-0.05	0.4
**WBC (k/μl)**	170	4.7 (3.8–6.1)	103	6 (4.7–8.0)	0.67	0.36	**9.01E-10**
**SLEDAI**	169	2	102	0	**3.30E-03**	-0.25	**3.11E-05**
Discoid Rash	170	2.94%	103	0%	0.16		
Malar Rash	169	12.43%	103	7.77%	0.31		
Rash	169	13.61%	102	8.82%	0.33		
Secondary Sjogren's	170	17.06%	103	12.62%	0.39		
Arthritis	169	5.92%	102	8.82%	0.46		
Active Nephritis	170	12.94%	103	9.71%	0.56		
Hematuria	169	1.18%	102	3.92%	0.2		
Proteinuria	169	4.73%	102	3.92%	1		
Pyuria	169	2.96%	102	6.86%	0.14		
**Alopecia**	169	10.06%	102	0.98%	**2.27E-03**		
Thrombocytopenia	169	3.55%	102	0%	0.09		
**Low Complement**	169	43.79%	102	19.61%	**4.30E-05**		
**Leukopenia**	169	6.51%	102	0.98%	**0.03**		
Anti-nuclear antibodies (ANA)	169	98.22%	103	94.17%	0.09		
Lupus anti-coagulant (LAC)	160	31.88%	91	34.07%	0.78		
Anti-ds DNA	170	69.41%	103	66.99%	0.69		
**Anti-Extractable nuclear Ags**	168	88.69%	103	68.93%	**9.46E-05**		

Represented p-values for comparison between groups. Spearman correlations (r_s_) and their p- value calculated for association of groups containing continuous variables with corresponding sLOX-1 levels. C3 = Complement 3, C4 = Complement 4, ESR = Erythrocyte sedimentation rate, WBC = White blood cells, SLEDAI = SLE Disease Activity Index.

### sLOX-1 is associated with inflammatory CVD risk in SLE patients

Stratification using sLOX-1 levels showed no difference in the 10-year risk for cardiovascular disease based on traditional Framingham Risk Scores (FRS) calculated using lipid risk factors (high sLOX-1 3.623 ± 0.5456 versus low sLOX-1 3.456 ± 0.3062) (Fig 1D and Table 2). Neither HDL nor LDL levels varied between the two groups (Table 2). Elevated hsCRP levels in the pathogenesis of atherosclerotic vascular disease is a hallmark of chronic inflammation and large-scale clinical trials have used hsCRP greater than or equal to 2 mg/L for defining an increased risk of CVD [33]. High sensitivity C-reactive protein (hsCRP) levels in the high sLOX-1 group (5.936 ± 1.362 mg/L; p<0.00001) were significantly higher than the low sLOX-1 group (2.187 ± 0.456 mg/L) (Fig 1E). The median hsCRP values for high sLOX-1 SLE patients was 2 mg/L compared to 1.3 mg/L in the low sLOX-1 patients (Table 2). Multivariate regression analysis adjusting for FRS did not change the positive correlation between sLOX-1 and hsCRP (β = +0.25; p = 3.07e^-05^) (Table 2), indicating that age, sex, systolic blood pressure, hypertension, smoking, diabetes, total cholesterol, HDL cholesterol and BMI did not account for the association of higher sLOX-1 levels with hsCRP. Besides hsCRP levels, triglycerides were increased in the high sLOX-1 group (136.3 ± 7.298, n = 99 versus 113.5 ± 4.699 mg/dL for low sLOX-1; p = 0.0095) and correlated significantly (r_s_ = +0.21; p = 0.0004) with sLOX-1 levels, even after adjustment for FRS (S2 Table).

**Table 2:** Association between sLOX-1 and cardiovascular risk factors.

Observation	Low LOX-1 (n)	Low LOX-1 (%/Median (IQR))	High LOX-1 (n)	High LOX-1 (%/Median (IQR))	P value	Spearman Correlation (r_s_)	P value (r_s_)
					(Group comparison)		
Hyperlipidemia	163	7.36%	99	2.02%	0.09		
Type-2 diabetes	163	4.29%	99	7.07%	0.4		
Statin use	163	13.50%	99	23.23%	0.06		
Smoker (Y/N)	163	4.29%	98	8.16%	0.27		
BP treated with medication	163	19.02%	99	28.28%	0.09		
**BP Diastolic**	163	71 (64–79)	99	68 (63–76)	**0.04**	-0.04	0.52
BP Systolic	163	121 (111–129)	99	121 (109–130)	0.77	0.01	0.87
Weight (Kg)	163	67.2 (55.9–87.4)	99	70 (55.9–87.4)	0.06	0.15	**0.01**
Height (CM)	163	159 (156.5–165.4)	99	160.5 (156.5–165.4)	0.22	0.05	0.39
BMI	163	25.79 (22.6–29.8)	99	27.71 (22.6–34.1)	0.07	0.15	**0.02**
Total cholesterol [mg/dL]	163	167 (138–193)	99	178 (153–204)	0.09	0.17	**0.01**
LDL [mg/dL]	163	81 (66–97)	99	87 (68–113)	0.07	0.15	**0.01**
FRS Lipids 10-Year Risk	163	2.4 (1.1–4.2)	98	1.8 (0.8–3.6)	0.11	-0.05	0.39
HDL [mg/dL]	163	59 (47–75)	99	57 (47–69)	0.62	-0.01	0.84
**Triglycerides [mg/dL]**	163	92 (73–137)	99	122 (75–166)	**0.02**	0.22	**4.00E-04**
**hsCRP (mg/dL)**	162	1.3 (0.7–2.9)	97	2 (1.0–5.5)	**0.01**	0.25	**3.07E-05**
Adjusted						**β value**	**P value**
**hsCRP (mg/dL)**	162	1.3(0.7–2.9)	97	2 (1.0–5.5)	**0.01**	0.25	**3.51E-05**

Multivariate regression analysis for hsCRP and sLOX-1 adjusting for FRS lipids. Adjusted correlation represented as β coefficient (Regression coefficient post standardization of variables dependent and independent variables). BP = blood pressure, hsCRP = high sensitivity C-reactive protein, BMI = Body Mass Index.

### High sLOX-1 is associated with normal complement levels and lower SLE disease severity

**Fig 2:**
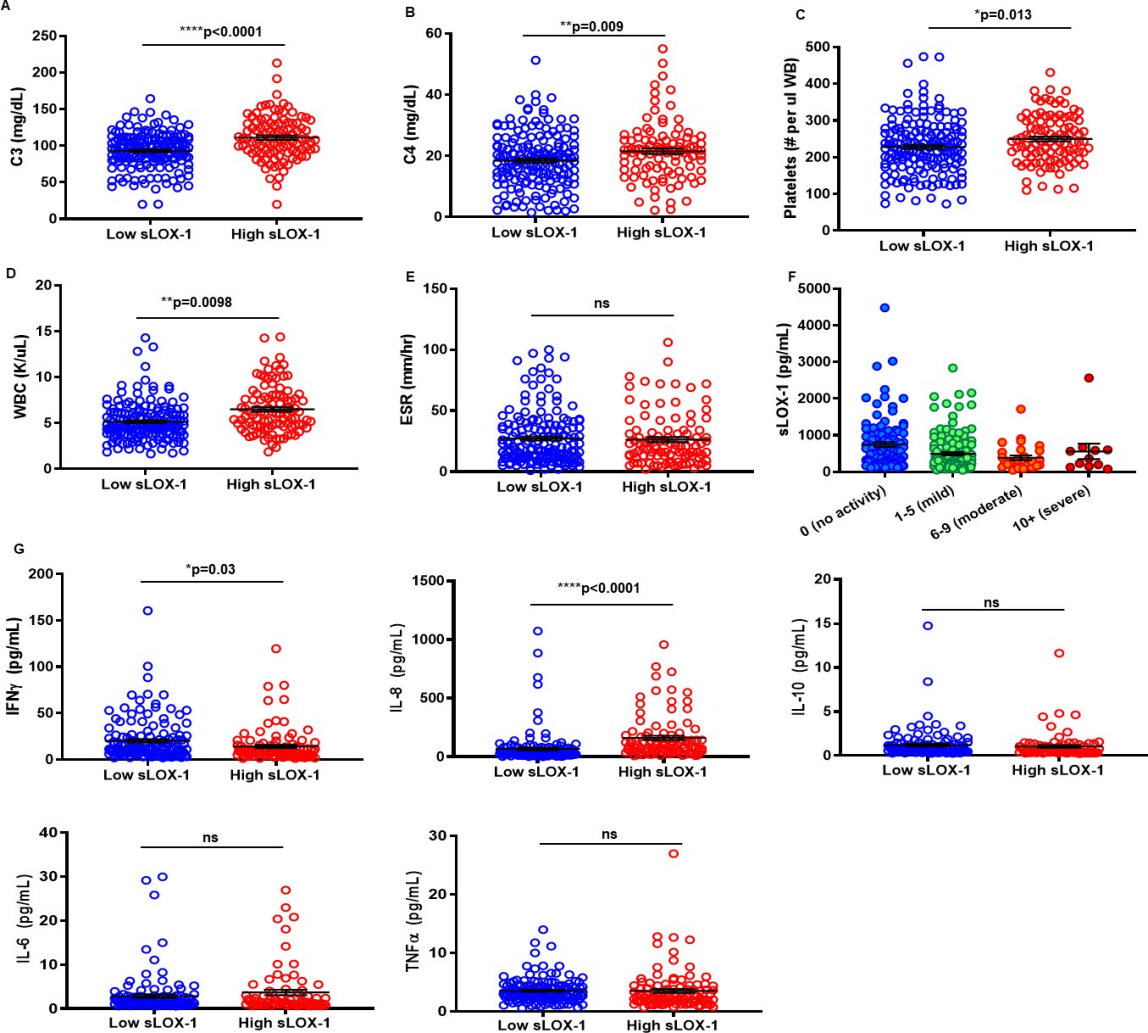
Relationship between sLOX-1 and SLE-related complement levels, platelets, WBC, erythrocyte sedimentation rate (ESR), SLEDAI scores and cytokines. Stratification of lupus clinical and serological parameters based on low and high sLOX-1 levels. Measurement of Complement (A) C3 mg/dL (93.12 ± 1.873, n = 171 versus 111.1 ± 2.95, n = 102) and (B) C4 mg/dL (18.48 ± 0.69, n = 171 versus 21.53 ± 0.9336, n = 102), (C) platelets (228.1 ± 5.718 (#/uL whole blood), n = 171 versus 249.7 ± 6.524, n = 103), (D) WBC (5.157 ± 0.1567 (K/uL), n = 170 versus 6.51 ± 0.2547, n = 102), (E) ESR mm/hr (27.21 ± 1.689, n = 169 versus 26.33 ± 2.127, n = 101) low sLOX-1 versus high sLOX-1 mean and SEM. (F) sLOX-1 levels binned by SLEDAI score. Bars represent mean±SEM sLOX-1 values for no (745±67.95 n = 104), mild 495±46 n = 127), moderate (383.4±65.41 n = 29) and severe (558.1±210.9 n = 11) disease activity. (G) Cytokines IFN-γ, IL-8, IL-10, IL-6 and TNF-α were measured by multiplex MSD ELISAs from SLE patients in the low sLOX-1 (n = 141) and high sLOX-1 (n = 101) groups. * p<0.05, ** p<0.01, ***p<0.001 and ****p<0.0001.

Laboratory tests to detect specific autoantibodies directed against nuclear or cytoplasmic antigens (serum anti- nuclear antigens (ANA), dsDNA, and extractable nuclear antigens (ENA)) were analyzed in the two sLOX-1 groups. Fewer patients were seropositive for ENA, which measures antibodies to saline-extracted antigens, anti-RNP, anti-SmRNP, anti-Ro, anti-La, anti-Sm, Scl-70, anti-Jo-1, in the high sLOX-1 group versus low sLOX-1 group, and there was a similar trend for ANA and anti-dsDNA. High sLOX-1 patients also had lower prevalence of hypocomplementemia (Table 1) and higher C3 and C4 levels in comparison to patients with low sLOX-1 (Fig 2A and 2B and Table 1). Complement levels also show moderate positive association with sLOX-1 as measured by correlation r_s_ = +0.36 (p = 1.30e^-09^) (Table 1). Platelets and white blood cell (WBC) counts were increased in the high sLOX-1 group (Fig 2C and 2D and Table 1). Erythrocyte sedimentation rate (ESR) which is an indicator of inflammation in SLE is comparable in the two comparison groups (Fig 2E). Interestingly, patients with low disease activity (Fig 2F) exhibited significantly higher sLOX-1 levels compared with mild and moderate disease patients. In support of this observation, a negative correlation was observed between sLOX-1 levels and SLEDAI scores (Table 1; r_s_ = -0.25, p = 3.11e^-05^). This is consistent with lower frequencies of autoantibodies, normal complement and cell counts in the high sLOX-1 patients. There was no evidence to suggest that the sLOX-1 levels were influenced by medications such as oral corticosteroids, hydroxycholoroquine, azathioprine, mycophenolic acid, methotrexate or biologics- usage (S3 Table). Although high sLOX-1 correlated with high hsCRP, sLOX-1 levels did not positively associate with individual clinical manifestations, overall disease activity, medications or hematological abnormalities.

### High sLOX-1 SLE patients have higher IL-8 levels and lower IFN-γ levels

We analyzed the levels of cytokines previously reported in SLE patients. High sLOX-1 patients had significantly enhanced IL-8 levels (163.3 ± 18.4 pg/mL, n = 101 p<0.0001) versus low sLOX-1 patients (68.83 ± 11.9 pg/mL, n = 141) (Fig 2G). IL-8 is downstream of LOX-1 and is secreted upon LOX-1 activation by ligands like CRP [34]. Thus, high sLOX-1 group having high IL-8 levels is in agreement with mechanism of LOX-1 activation. Interestingly, lower IFN-γ levels associated with high sLOX-1 patients (14.25 ± 1.9 pg/mL, n = 101 p = 0.03) versus low sLOX-1 patients (20.25 ± 1.9 pg/mL, n = 141). IL-10, IL-6 and TNF-α levels remained unchanged between the two groups (Fig 2G). In the light of previous reports that have shown a poor correlation between IL-8 and disease severity [35], our observation that sLOX-1 can be used to define an SLE population with significantly high IL-8 levels is noteworthy.

### Patients with high sLOX-1 have impaired HDL functionality, high proinflammatory HDL and high oxidized LDL levels

**Fig 3:**
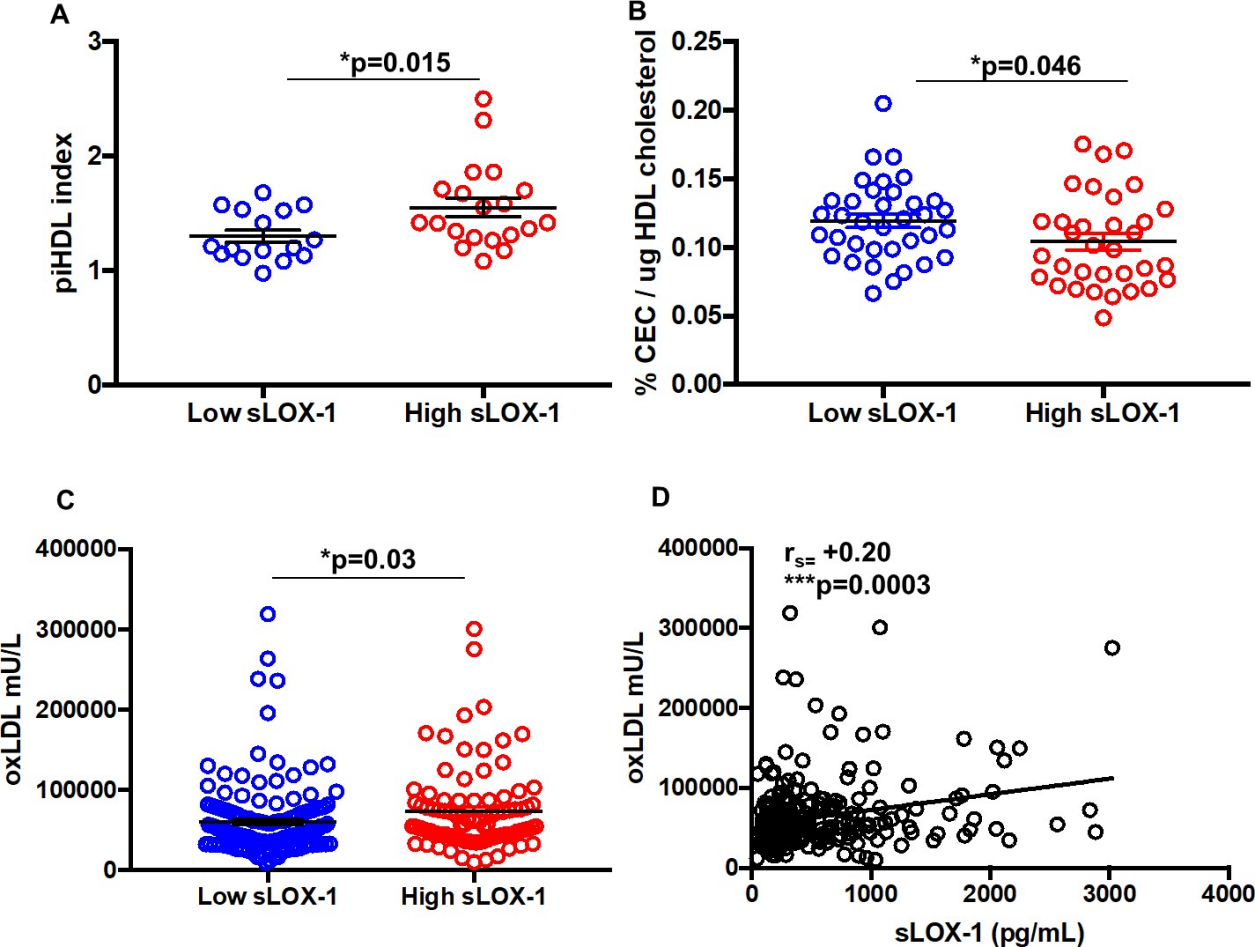
Assessment of lipid function in low and high sLOX-1 SLE patients. (A) Increased proinflammatory HDL (piHDL) in high sLOX-1 (n = 20) SLE patients versus low sLOX-1 (n = 16) SLE patients using DCFDA as a substrate to measure LDL oxidation; (B) Cholesterol efflux capacity of SLE HDL from donors with low sLOX-1 (n = 36) and high sLOX-1 (n = 33). Global cholesterol efflux capacity (CEC) was normalized to total HDL cholesterol quantification from each donor before being matched with sLOX-1 levels. (C) Levels of oxLDL in high (72902 ± 5345 mU/L, n = 93) and low sLOX-1 SLE patients (59489 ± 3396 mU/L, n = 163). (D) Spearman correlation (r_s_) between oxLDL and sLOX-1. Data represented as mean ± SEM. * p<0.05, ** p<0.01, ***p<0.001 and ****p<0.0001.

In a study on SLE individuals, 86.7% of patients with atherosclerotic plaques had increased piHDL plasma levels compared with 40.7% of those without plaques, suggesting that piHDL reflects increased CVD risk [9]. We determined piHDL index, which is a measure of the ability of HDL to inhibit LDL oxidation. HDL from high sLOX-1 patients had an index of 1.552 ± 0.08214 (p<0.015), compared to 1.3 ± 0.05396 in the low sLOX-1 patients (Fig 3A). Further, we confirmed previous published studies indicating that proinflammatory SLE HDL, functions as a ligand for LOX-1 [24], inhibiting nuclear translocation of the anti-inflammatory transcription factor ATF3 in macrophages, an effect reversed with an LOX-1 receptor blocking antibody (S1 Fig).

In further studies of HDL functionality, we evaluated the ability of SLE or healthy HDL to accept cholesterol particles from macrophages in CEC assays. We found that CEC in the high sLOX-1 SLE HDL (0.104 ± 0.005856%) was impaired compared to low sLOX-1 SLE HDL (0.1193 ± 0.004774%; p = 0.04) when normalized to HDL cholesterol content (Fig 3B). In addition, we observed that the plasma level of oxidized LDL (oxLDL) was significantly higher in high sLOX-1 patients (72902 ± 5345 mU/L) compared to low sLOX-1 patients (59489 ± 3396 mU/L, p = 0.03) (Fig 3C). These findings are consistent with previous reports showing that both piHDL and oxLDL, which function as ligands for the LOX-1 receptor are elevated in the plasma of SLE patients compared to controls [9]. These data show for the first time that a significant positive correlation exists between oxLDL levels and sLOX-1 levels in SLE patients (r_s_ = +0.23, p<0.0003), even after adjusting for FRS in multivariate regression analysis (Fig 3D and S2 Table). Interestingly, neither age, CEC efflux or oxLDL showed differences when patients were grouped based on high or low hsCRP levels (S2 Fig). This suggests that sLOX-1 is a more sensitive measure of distinguishing an SLE population with dysregulated lipoprotein that is responsible for a higher risk for CVD related events.

### LOX-1 signaling on monocytes drives proinflammatory cytokine secretion

**Fig 4:**
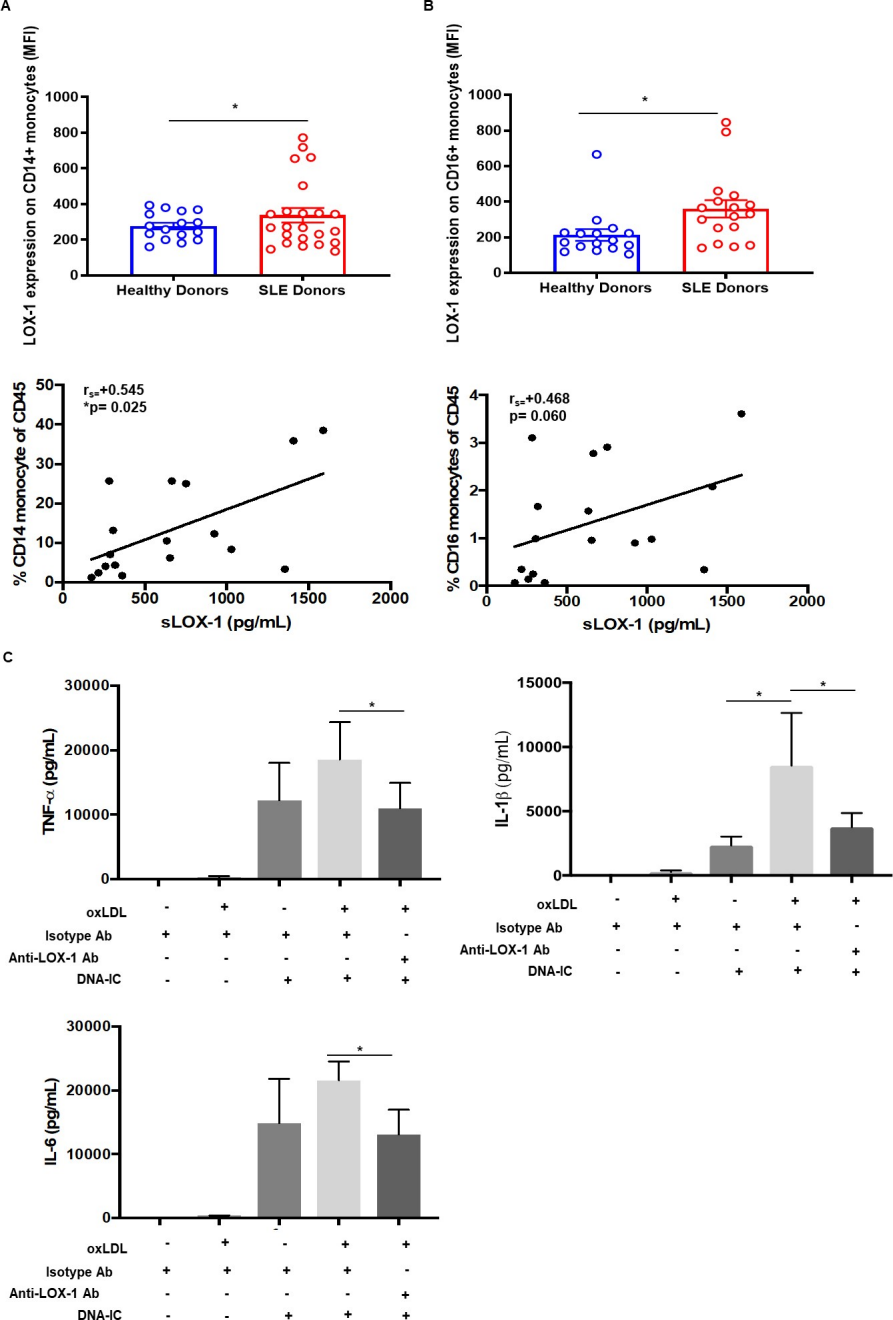
Flow cytometric assessment of LOX-1 on SLE monocytes and oxLDL-induced proinflammatory cytokine secretion from healthy monocytes. Flow cytometric analysis was performed on PBMCs from SLE patients (n = 17) and healthy individuals (n = 15) for LOX-1 expression. After gating out T cells, B cells and granulocytes, (A) and (B) CD14+/CD16+ staining was used to determine LOX-1 expression on monocytes (top panel). Spearman correlation (r_s_) between numbers of CD14+ and CD16+ monocytes and sLOX-1 (bottom panel). (C) Cytokine secretion measured using MSD-based immunoassay depicts TNF-α, IL-1β and IL-6 levels upon oxLDL (30 μg/mL) priming for 3h, followed by DNA-IC (20 μg/mL) stimulation by itself and in combination for 24h. n = 4. Data represented as mean ± SEM. * p<0.05, ** p<0.01, *** p<0.001 and **** p<0.0001.

Next, we examined membrane LOX-1 expression on multiple immune cell subsets in SLE and healthy donors. Both classical (CD14^+^) and non-classical (CD16^+^) monocytes had significantly higher levels of membrane LOX-1 expression compared to healthy donors (Fig 4A and 4B top panel). Numbers of CD14^+^ and CD16^+^ monocytes correlated positively with sLOX-1 levels (Fig 4A and 4B bottom panel). Phenotypic characterization of other myeloid phenotypes, inflammatory monocytes (CD14^+^/CD16^+^) and dendritic cells (mDC1 and mDC2) did not reveal elevated LOX-1 or any correlation between sLOX-1 and cell counts (S3 Fig). Functionally, priming CD14+ monocytes from healthy individuals with oxLDL (3h) enhanced their response to DNA-immune complex (DNA-IC stimulation) (24h) (Fig 4C). Upregulation of major cardiovascular disease related proinflammatory cytokines, TNF-α, IL-1β and IL-6, were inhibited with anti-LOX-1 receptor antibody treatment. This data indicates that oxLDL drives proinflammatory signaling through the LOX-1 receptor.

### Cleaved sLOX-1 levels strongly correlate with TACE activity in SLE patients

**Fig 5:**
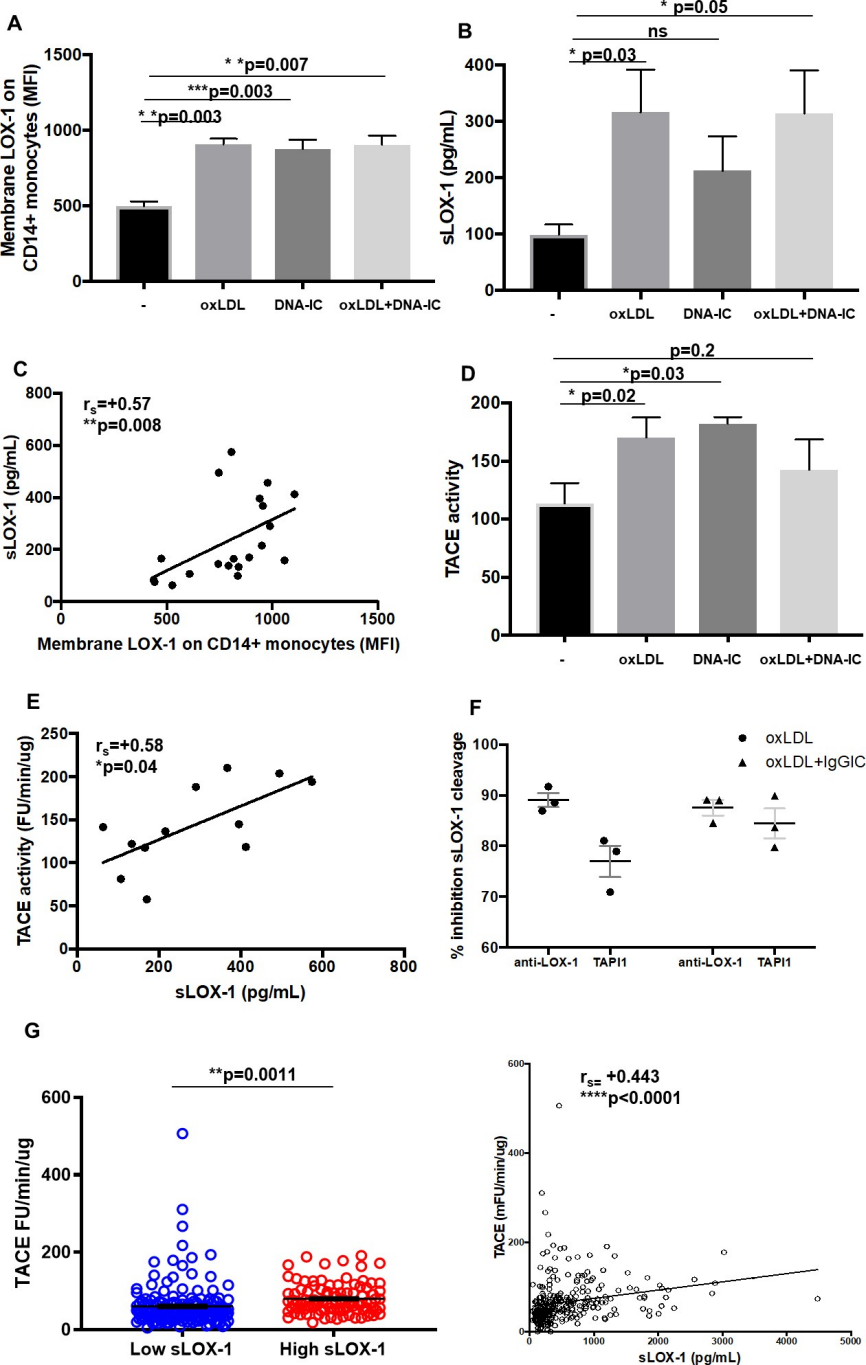
Relationship between induction of membrane LOX-1/sLOX-1 cleavage with TACE activity. Healthy monocytes were isolated from PBMCs and treated with oxLDL (30 μg/mL) for 3h followed by DNA-IC (20 μg/mL) (co)stimulation for 24h. (A) LOX-1 (MFI) expression on CD14+ monocytes. (B) sLOX-1 (pg/mL) detection from supernatant post 24h treatment. (C) Spearman correlation (r_s_) between membrane LOX-1 (MFI) and sLOX-1 (pg/mL). (D) Tumor necrosis factor alpha activating enzyme (TACE) activity (FU/min/μg) was measured using a fluorogenic peptide substrate (Mca-PLAQAV-Dpa-RSSSR-NH2) from supernatants post 24h treatment. (E) Spearman correlation (r_s_) between sLOX-1 (pg/mL) and TACE activity (FU/min/μg). Data from *in vitro* experiments represented as mean ± SEM. (F) Inhibition of sLOX-1 cleavage in the presence of either the TAPI-1 inhibitor (100 μM) or the LOX-1 receptor blocking antibody. (G) TACE activity measurements normalized to total protein in SLE patients with low (2.741 ± 0.3491, n = 168) and high (5.134 ± 0.7696, n = 100) sLOX-1. Relationship between TACE activity (FU/min/μg) and sLOX-1 (pg/mL) from matched patient serum shown by Spearman correlation (r_s_). Data represented as mean ± SEM. * p<0.05, ** p<0.01, *** p<0.001 and **** p<0.0001.

To determine if membrane and sLOX-1 are concordantly induced, we assessed their levels following stimulation with oxLDL and/or DNA-IC. Monocytes increased membrane and soluble LOX-1 upon oxLDL, DNA-IC and ox-LDL + DNA-IC stimulation at 24h (Fig 5A and 5B). A strong correlation was found between membrane and soluble LOX-1 levels (r_s_ = +0.57, p = 0.008), indicating that the source of sLOX-1 may be membrane LOX-1 (Fig 5C). Previously, LOX-1 has been shown to be cleaved by ADAM activity [30, 31]. To determine if this is the case, we assessed whether activity of TNF-α converting enzyme (TACE) is increased upon stimulation with oxLDL, DNA-IC and oxLDL+DNA-IC (Fig 5D). Following stimulation of monocytes, TACE activity positively correlated with sLOX-1 levels in culture supernatants (Fig 5E). In addition, sLOX-1 cleavage was inhibited in the presence of either TACE inhibitor (TAPI-1, 100 μM) or 50 μg/mL of the LOX-1 receptor blocking antibody (Fig 5F). The physiologic relevance of this relationship was also observed in SLE patients, where TACE activity levels were higher in the high sLOX-1 groups (low sLOX-1 mean = 60.4 ± 4.409, n = 167, high sLOX-1 mean = 79.75 ± 3.851, n = 101) and correlated significantly with sLOX-1 levels (r_s_ = 0.422, p = 3.01e^-13^) (Fig 5G and S2 Table). This data indicates that upregulation of surface LOX-1 and TACE activity lead to increased sLOX-1 levels in SLE patients.

### oxLDL promotes low-density granulocytes to form extracellular traps

**Fig 6:**
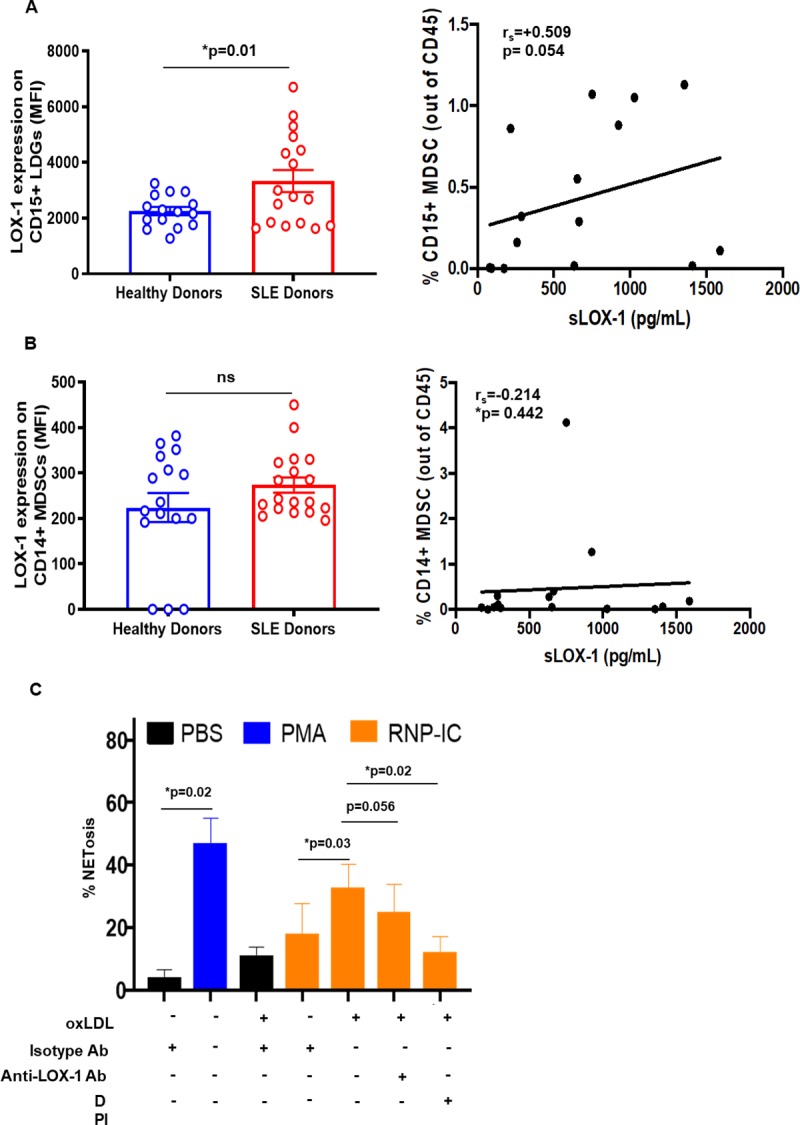
Flow cytometric and functional analysis of low-density granulocytes (LDGs) from SLE patients. Flow cytometric analysis was performed on PBMCs from SLE patients (n = 17) and healthy individuals (n = 15) for LOX-1 expression. After gating out CD3^+^/CD19^+^/CD20^+^/CD56^+^/HLADR^+^ cells, CD33^+^ and CD11b^+^ cells were separated based on CD14 and CD15 expression. Membrane LOX-1 (MFI) levels were determined in (A) CD14^+^ monocytic myeloid derived suppressor cells (MDSCs) and (B) CD15+ LDGs. (C) NET generation (MPO-red dye) from CD15^+^ LDGs following 3h oxLDL pre-treatment and stimulation with patient-derived RNP-immune complexes for 4h was observed. n = 4. Data represented as mean ± SEM. * p<0.05.

Low density granulocytes (LDGs) are prevalent in SLE patients but not healthy controls [36], and these cells are sensitive to the generation of NETs which can induce endothelial injury and promote inflammatory responses [37]. Interestingly, we found that LDGs (which have similar phenotype to PMN- myeloid derived suppressor cells (MDSCs) [38, 39] expressed high levels of LOX-1 in SLE patients, and slightly lower levels in healthy donors (Fig 6A). LOX-1 expression on CD14+ monocytic MDSCs were much lower and there was no difference between SLE and healthy donors (Fig 6B). The increased frequency of LDGs (S3 Fig) in SLE permitted functional examination of the effect of oxLDL on NET formation. RNP immune complexes (RNP-IC) from SLE patients have been shown to trigger the generation of NETs [40]. Stimulation of SLE patients’ LDG with oxLDL enhanced RNP-IC induced generation of NETs (Fig 6C). The impact of oxLDL on NET formation was inhibited by the anti-LOX-1 receptor blocking antibody or a ROS inhibitor (DPI; 10 μM).

## Discussion

We report for the first time that sLOX-1 may be useful in identifying SLE patients with cardiovascular risk. sLOX-1 levels were two-fold higher in SLE patients. We postulate that sLOX-1 is driven by activation of inflammatory pathways associated with either SLE disease activity or atherogenesis secondary to SLE diagnosis. In our studies, sLOX-1 levels were positively associated with hsCRP levels, proinflammatory HDL, oxLDL and impaired HDL efflux, rather than traditional risk factors and SLE disease activity.

Although the mechanistic explanation for the dissociation between serum sLOX-1 levels and SLE disease activity require further studies, our data suggests that different aspects of inflammation contribute to SLE and CVD. Consistent with our findings, in an independent cohort of SLE patients with low disease activity and without pre-existing CVD, preclinical vascular damage was observed and associated with type I IFN activity [41]. One of the largest studies to examine cardiovascular risk in SLE was conducted on 1,784 patients with a total of 9,485 person-years follow-up showed cardiovascular risk was associated with current rather than past disease activity, and CV events may be precipitated by acute changes in disease activity [42]. This is not necessarily discordant with our data since the sLOX-1 levels may reflect underlying vascular inflammation.

It was also notable that the patients with high sLOX-1 levels were younger than those with low sLOX-1 levels. Both groups had a similar time since diagnosis, which indicates that the high sLOX-1 group also had an earlier onset of disease. This may appear unexpected since oxLDL and LOX-1 increase with age and cardiovascular disease in the general population. However, SLE patients have a greater risk of cardiovascular disease and the disparity is particularly evident in young females. Women under the age of 44 with SLE have >52-fold risk of myocardial infarction. Coronary events are rare in women under 55 years, whereas 54% of cardiac events in a female SLE patient population occurred under the age of 44 [43]. Therefore, the increased sLOX-1 levels in this younger SLE population may reflect subclinical atherosclerosis and the enhanced risk of CVD which is consistent with high sLOX-1 levels associated with higher hsCRP, oxLDL and impaired HDL functionality.

The MDC cohort enabled the first large, prospective study showing relevance for high levels of sLOX‐1 with plaque size and increased risk for future ischemic stroke. An analysis of stroke risk factors in the MDC cohort on 4703 subjects showed that increasing sLOX-1 levels in plasma correlated significantly with increases in hsCRP and size of carotid plaques. A separate CPIP (Carotid Plaque Imaging Project) cohort further validated significant correlation between plaque LOX-1 and sLOX-1. Furthermore, proinflammatory cytokines and ox-LDL levels correlated with plaque as well as soluble LOX-1 content [32]. In a recent study with 173 psoriasis patients by Dey et al., sLOX-1 was shown to be correlated with non-calcified plaque burden irrespective of hyperlipidemia. Additionally, an improvement in psoriasis was associated with a decrease in sLOX-1 levels [29].

Our data showed that the SLE patients with high sLOX-1 levels had significant increase in hsCRP, a well-known clinical marker of inflammation in cardiovascular disease [44]. hsCRP has been successfully used as a patient stratification marker for ACS and reduction in hsCRP has been shown to result in CV event reduction [33, 45, 46]. sLOX-1 is elevated in the systemic and coronary circulation of patients with acute coronary syndrome (ACS) [25, 26] and is proposed as a marker for presence of active and vulnerable inflammatory atherosclerotic lesions [47]. A recent “liquid biopsy” analysis of proteins released locally by the vasculature indicated that LOX-1 is the most abundant protein released by vessels with atherosclerotic plaques compared to controls [48].

Based on evidence from our current study, we postulate that even in low disease activity patients, sLOX-1 may be indicative of low-grade inflammation contributing to atherogenesis or subclinical atherosclerosis. sLOX-1, in fact, performed better than hsCRP in predicting higher oxLDL levels and impaired CEC in SLE patients (Fig 3 and S1 Fig). It has been apparent that CRP is not a reliable predictive marker of cardiovascular disease or underlying inflammation in SLE patients as it is in other conditions. This likely reflects the increased type I IFN activity in SLE patients which has been shown to suppress CRP levels [49]. Given this limitation of CRP as a CV marker for SLE patients, sLOX-1 may serve as a better predictive marker of dyslipidemia and cardiovascular risk. Longitudinal studies in SLE cohorts will be required to confirm this finding.

Dysregulated lipoproteins also represent a significant family of atherogenic LOX-1 ligands and are usually a hallmark of CVD inflammatory disease. piHDL is the HDL incapable of removing reactive oxygen species (ROS) from LDL [10]. In our high sLOX-1 group, we observed higher piHDL levels, a measure that McMahon et al. established as a 28-fold increased risk predictor of plaque deposition [50]. 50% of women with SLE had piHDL, as compared with fewer than 10% of age-matched healthy women and 87% of SLE patients with plaque on carotid ultrasound had piHDL, as compared with 41% of those without carotid plaque. Normal HDL also functions in maintaining efficient cholesterol efflux, a mechanism that regulates reverse cholesterol transport. In light of multiple failures in past trials involving HDL cholesterol-raising therapies [51], a large-scale longitudinal study following 2924 individuals over 9.4 years was undertaken to demonstrate an inverse relationship of HDL cholesterol efflux capacity (CEC) with the incidence of cardiovascular events [52, 53]. In SLE, ATP-binding cassette A1 and G1 (ABCA1/ABCG1)-dependent CEC has been shown to be impaired [15, 54]. In our study, high sLOX-1 patients were significantly impaired in ABCA1-dependent CEC compared to the low sLOX-1 patients. Based on our data, sLOX-1 levels can distinguish a subset of SLE patients with the likelihood of having impaired HDL cholesterol, and potentially increased oxidized LDL (oxLDL) levels.

Atherosclerotic lesions develop when low-density lipoproteins (LDLs) are oxidized into oxLDL phospholipids by ROS generation from endothelium dysfunction [55] combined with the inability of HDL to inhibit their oxidation. oxLDL is then phagocytized by macrophages leading to the formation of foam cells that necrotize and give rise to atherosclerotic plaque [56]. Our studies for the first time establish that higher oxLDL levels are prevalent in SLE patients with high sLOX-1 and that oxLDL levels positively and significantly correlate with sLOX-1 levels. Mechanistically, higher levels of oxLDL can potentiate increased activation of LOX-1 pathway.

oxLDL engages in a positive feedback loop enhancing LOX-1 levels on macrophages and endothelial cells [57]. We detected significantly upregulated LOX-1 on the surface of monocytes and LDGs in SLE patients with their numbers strongly correlating with sLOX-1 levels. Our *in vitro* studies showed that oxLDL can induce membrane LOX-1 expression on monocytes and further drive cleavage of sLOX-1 in a TACE-dependent manner. Both LOX-1 blocking antibody and the TACE inhibitor, TAPI-1, could reverse sLOX-1 cleavage. This is consistent with previous studies on human monocyte-derived macrophages where sLOX-1 released by CRP stimulation has been shown to be attenuated by a TACE inhibitor or TACE siRNA [31]. TACE activity in human plasma is measurable [58, 59]. Detection of TACE activity in serum of SLE patients primarily, revealed a strong positive correlation of TACE activity with sLOX-1 levels.

Functionally, oxLDL was pathogenic to monocytic cells through its ability to prime them to be more proinflammatory with DNA-IC stimulation. We found that co-stimulation with DNA-IC leads to TNF-α, IL-1β and IL-6 secretion that can potentially feed forward into the LOX-1/sLOX-1 induction pathway since proinflammatory cytokines such as TNF-α and IL-1β have been shown to induce LOX-1 expression [60, 61]. Breaking this feed forward loop can be beneficial, for example, IL-1β β blockade (CANTOS trial) has been shown to successfully reduce inflammation-driven CVD [62, 63].

LOX-1 was also increased on CD15^+^/HLADR^-^ LDGs also known as granulocytic MDSCs, a specialized subset of polymorphonuclear cells found to be elevated in SLE, cancer and inflammatory diseases [64–66]. Our data reveals that oxLDL can prime LDGs in a LOX-1-dependent manner to promote the generation of NETs. This process typically yields the release of DNA, myeloperoxidase (MPO), neutrophil elastase (NE), proteinase 3 (PR3); secondary neutrophil granules: lactoferrin, pentraxin 3 and gelatinases [67]. The presence of NETs has been documented in human plaques with a superficial erosion-like morphology [68]. Plasma from patients with eroded plaques exhibit high MPO, produced primarily by neutrophils, compared with those with ruptured lesions [69]. In SLE patients with plaques, LDG-specific NET associated gene signatures, including MPO, correlated with noncalcified plaque burden [70]. In fact, LDGs through NET formation were also shown to enhance HDL oxidation, implying loss of atheroprotective capacity [15]. Thus, NET-induced HDL oxidation [70] can convert HDL into a LOX-1 activating ligand [24]. Since our studies also show that LOX-1 activation with oxLDL promotes the generation of NETs, it is conceivable that that NET-induced lipid oxidation may amplify LOX-1 mediated NET formation. These results justify additional studies to evaluate LOX-1-mediated LDG activation and NET generation in atherosclerotic conditions.

In this cross-sectional study, we have provided indirect evidence that sLOX-1 levels may represent a useful biomarker for cardiovascular risk in SLE patients. The size of the study was underpowered to provide direct linkage between LOX-1 levels and cardiovascular events. It was notable that 10 patients that were identified with a prior or recent history of cardiovascular disease trended towards higher LOX-1 levels 750.3± 742.9 pg/mL compared to the SLE population as a whole 580.9 ± 36.1 pg/mL. In lieu of a longitudinal study to evaluate soluble LOX-1 levels, flares in disease activity and the development of cardiovascular disease, a study has been initiated to evaluate soluble LOX-1 levels and sub-clinical atherosclerosis in SLE patients. Prior studies of this sort have indicated that sLOX-1 levels correlate with carotid plaque inflammation and risk of ischemic stroke [32], and track with the burden of non-calcified coronary plaques in psoriasis patients [29].

We have provided insight into the regulation and LOX-1 expression, and experimental evidence which indicates that oxidized lipids may drive LOX-1 dependent atherogenic pathways in SLE patients. Perturbed activation of the LOX-1 pathway may explain the increased risk of cardiovascular events in SLE patients, and the inhibition of the LOX-1 pathway may provide protection from the development of cardiovascular disease.

## Materials and methods

### Patients samples and clinical assessments

SLE patient blood and serum samples were obtained from the Warren G. Magnuson Clinical Center Blood Bank (Bethesda, MD) as approved by the National Institute of Arthritis and Musculoskeletal and Skin Diseases/National Institute of Health between 2013 and 2018. Clinical and demographic characteristics, SLEDAI-2K [71] and Framingham Risk Scores (FRS) were calculated at each visit. Laboratory parameters including fasting blood glucose and lipid panel, white blood count, platelet count, C3, C4 complement levels, and systemic inflammatory markers such as hsCRP and ESR were quantified in the clinical laboratory at the NIH. Laboratory tests for anti-nuclear antibodies (ANA), extractable nuclear antigens (ENA) and double-stranded DNA (dsDNA) antibodies were all performed and reported for the cohort. Medication usage for all patients was also noted. Blood and serum from healthy donors were obtained from individuals enrolled at the MedImmune Research Specimen Collection Program. SLE and healthy cohorts are described in S1 Table.

### Measurement of sLOX-1 levels in SLE serum and supernatants

An in-house sandwich ELISA was developed using the Mesoscale diagnostics (Mesoscale Diagnostics, MD, USA) platform to measure soluble LOX-1 levels in human serum and supernatants. MSD high bind plates were coated with 5 μg/ml in-house generated anti-LOX-1 antibody overnight, blocked for 1 hour and samples (25 μl/well, no dilution) were added along with recombinant human LOX-1 as standard for 2h. Plates were washed using MSD Tris wash buffer 3 times after each incubation step. Human LOX-1/OLR1 antibody (AF1798 from R&D systems, MN, USA) was sulfotagged using MSD conjugation kit (R31AA-2) to generate detection antibody. Sulfo-tagged detection antibody was added and incubated for 1 hr. 2X MSD read buffer was used to read plates on MSD machine. sLOX-1 levels from the samples were interpolated from standard curve values using MSD workbench software (Mesoscale Diagnostics, MD, USA).

### HDL assays

HDL was isolated from human serum by polyethylene glycol precipitation (PEG) of LDL using 20% w/v PEG in PBS as described before [72].

#### Proinflammatory HDL (piHDL) index

piHDL was measured using a cell-free assay has been developed and reported previously [9, 73, 74]. 20 μL of the normal LDL (Cell Biolabs, CA, USA) at a concentration of 50 μg/ml and 90 μL of test HDL from healthy of SLE individuals at a final concentration of 10 μg/mL cholesterol were incubated in quadruplicate in 96-well plates for 1h. 10 μL of DCFH-DA solution (0.2 mg/mL) was then incubated in each well for 2 hours. Presence of oxidized form of LDL leads to the conversion of normally non-fluorescent dichlorofluorescein diacetate (DCFH-DA) into a fluorescent form (DCFH). DCFH is then measured on a plate reader (SpectraMax, Molecular Devices, CA, USA) at an excitation wavelength of 485 nm and an emission wavelength of 530 nm. Fluorescence units were then compared with absence of test HDL in the mixture which was set at a value of 1.

### Cholesterol Efflux Capacity (CEC) assays

CEC assays were performed based on previously reported technique on J774 cells [75]. Briefly, cells were plated and radiolabeled with 2 μCi of ^**3**^H-cholesterol/ml. 0.3 mmol/l 8-(4-chlorophenylthio)-cAMP was added to the cells for 16h to upregulate ATP-binding cassette transporter A1 (ABCA1). Serum HDL obtained after apoB depletion, as described above, from healthy and SLE individuals was added for 4h. Liquid scintillation counting was performed to count effluxed radioactive cholesterol by HDL from cells. CEC was then calculated by using the following formula: (μCi of ^**3**^H-cholesterol in media containing 2.8% apoB-depleted subject plasma–μCi of ^**3**^H-cholesterol in serum-free media/μCi of ^**3**^H-cholesterol in media containing 2.8% apoB-depleted pooled control serum–μCi of ^**3**^H-cholesterol in pooled control plasma-free media). % efflux was then divided by total cholesterol content from HDL serum that was added to obtain % efflux per μg HDL cholesterol. The pooled healthy serum was obtained from 3 healthy volunteers. All assays were performed in triplicate.

### oxLDL measurements

Oxidized LDL (oxLDL) was measured in human serum samples by an ELISA kit (Mercodia Inc, Uppsala, Sweden) which is a solid phase two-site enzyme immunoassay based on the direct sandwich technique in which two monoclonal antibodies were directed against separate antigenic determinants on the oxidized apolipoprotein-B molecule. During incubation, oxLDL in the sample reacts with anti-oxLDL antibodies bound to microtitration well. After washing, which removes non-reactive plasma components, a peroxidase conjugated anti-human apolipoprotein B antibody recognizes the oxidized LDL bound to the solid phase. After a second incubation and a simple washing step that removes unbound enzyme labeled antibody, the bound conjugate is detected by reaction with 3,3’, 5,5’-tetramethylbenzidine (TMB). The reaction is stopped by adding acid to give a colorimetric endpoint, then read spectrophotometrically.

### Immunophenotyping of SLE PBMCs

Human PBMCs (healthy and SLE) were isolated using BD Vacutainer®, CPT^™^ mononuclear cell preparation tubes (BD Biosciences, NJ, USA) by density gradient centrifugation at 1700 g, deceleration = 6, for 25 min at 25˚ C. PBMCs were obtained, washed two times and resuspended as 1x10^**6**^ cells per 100 μL in Brilliant Violet stain buffer (BD Biosciences, NJ, USA). Cells were Fc receptor blocked for 15 minutes (TrueStain FcX, Biolegend) on ice and stained with various antibody panels. Cells were then washed and fixed by resuspending in 1% PFA. Data was collected using a BD FACS LSRII and analyzed using FlowJo version 10 (FlowJo LLC, OR, USA). Cells were stained with anti- CD3/CD19/CD20/CD56/CD45 (Lin^**-**^) antibodies and further stained for CD7^**-**^/HLADR^**+**^/CD141^**+**^/CD11c^**+**^/CD14^**+**^/CD16^**+**^ to specifically define gates for monocytes and dendritic cells (mDC1 and mDC2). LDGs were identified as Lin^**-**^/ HLADR^**-**^/CD15^**+**^/CD11b^**+**^/CD33^**+**^/CD14^**-**^/CD15^**+**^ and monocytic MDSCs stained as Lin^**-**^/ HLADR^**-**^/CD15^**+**^/CD11b^**+**^/CD33^**+**^/CD14^**+**^/CD15^**-**^. All cell types were stained for LOX-1.

### In vitro cytokine secretion assay from monocytes

Monocytes isolated from PBMCs of healthy individuals using the Robosep^™^ monocyte isolation kit (StemCell, MA, USA) were cultured as 500,000 cells per 250 μL X-VIVO 15 (Lonza, Basel, Switzerland) media supplemented with 10% FBS and 1% penicillin-streptomycin. Anti-LOX-1 antibody (50 μg/mL) or Control IgG antibody (Clone: NIP228) were used to pre-treat monocytes for 30 min at 37˚ C. Cells were then treated with 30 μg/mL oxLDL (Cell biolabs, CA, USA) for 3 h and then stimulated with DNA-immune complexes (1 μg/mL CG50 + 20 μg/mL E11 IgG antibody) for 24 h. Supernatants were then collected, diluted at 1:6 ratio and added to 7-Plex Human Proinflammatory cytokine plates (MesoScale Diagnostics, MD, USA) to detect various cytokines.

### Measurement of TACE activity in SLE serum and supernatants

TACE activity was measured in SLE patient serum as well as from supernatants of stimulated monocytes with oxLDL and DNA-IC. Where indicated, anti-LOX-1 antibody (50 μg/mL) or TAPI-1 inhibitor (100 μM) were used to pre-treat cells for 30m. TACE activity was measured by using a synthetic peptide substrate containing the cleavage site (Mca-Pro-Leu-Ala-Gln-Ala-Val-Dpa-Arg-Ser-Ser-Ser-Arg-NH2) primarily for ADAM17 and related enzymes such as ADAM8, ADAM9 and ADAM10 (R&D Systems, Inc., MN, USA). The cleavage site by ADAM17 and ADAM10 is the peptide bond between Ala and Val. 10 μL of each sample (serum or supernatant) was added to 90 μL of 25 mM Tris buffer, pH 8.0. The substrate was then added at a final concentration of 10 μM in a total of 100 μL reaction mixture for 1h. Fluorescence units (FU) were measured using a fluorescent microplate reader, Spectramax (Molecular Devices, CA, USA), with an excitation wave length at 320 nm and an emission wavelength at 405 nm. TACE activity results were normalized to total protein concentration of serum samples or supernatant. Enzymatic activity of TACE is represented as FU/min/μg.

### In vitro determination of NET formation by LDGs

Isolated LDGs at 2x10^4^ cells per well were plated in 96-well flat-bottom plates in HBSS (Ca^2+^ and Mg2+ free, ThermoFisher) with 2% heat-inactivated FBS. After incubation with anti-LOX-1 antibody (50 μg/mL) or diphenyleneiodonium (DPI; 10 μM) treatment at 37°C for 30 min, cells were primed with oxLDL where indicated. PMA (25 nM) or RNP immune complexes (RNP 1 μg/mL + 2% RNP^+^ SLE serum) were used to treat LDGs for 4 h in the presence of anti-MPO-FITC at 5 μg/mL and NucRed at 4 μL/100 μl/well. Without washing, cells were imaged using high content imaging system, Incucyte (Essen BioScience, MI, USA) with a 20x objective. Cells from 9 fields were quantified in duplicates for each treatment. The number of MPO positive NETs were quantified by the Incucyte software (Essen BioScience, MI, USA).

### Statistical analysis

Summary statistics are presented as mean ± SEM or median as indicated for continuous variables and categorical variables. For group comparisons, two-tailed Student’s t test was used for parametric data analysis as seen in *in vitro* data, and Welch’s test was used for nonparametric data analysis for comparing sample means with unequal variances and unequal sample sizes in low sLOX-1 versus high sLOX-1 SLE groups, when normal distribution of the data was not guaranteed. For correlation analysis, Spearman’s correlation analysis was used and the coefficient reported as r_**s**_. For certain values in tables, standardized multivariate regression analysis was performed, and standardized β-coefficients and p values were reported. hsCRP, TACE activity, triglycerides, HDL efflux, oxLDL levels represent dependent variables, adjusting for cardiovascular and cardiometabolic risk factors. Statistical analysis was performed using R. P values ≤ 0.05 are considered statistically significant.

## Supporting information

S1 Table Description of healthy and SLE cohort. (DOCX)

S2 Table Association between sLOX-1 (dependent variable) and triglycerides, oxLDL, CEC and TACE activity. Adjustment for FRS. Unadjusted/adjusted associations between sLOX-1 (dependent variable) and triglycerides, oxLDL, CEC and TACE activity. Adjustment for FRS. Observations that are in ‘bold’ denote significant findings.
(DOCX)

S3 Table Association between sLOX-1 and SLE medications. (DOCX)

S1 Fig SLE high sLOX-1 HDL inhibits nuclear co-localization of ATF3. (A) Confocal imaging of primary macrophages exposed to HDL from healthy donors and SLE high sLOX-1 patients. (B) Quantification of nuclear localization of inflammation resolution factor ATF3 in response to exposure to HDL, or blocking with anti-LOX-1 Ab prior to exposure to HDL.
(TIF)

S2 Fig High hsCRP levels do not help identify SLE patients with dysregulated lipoproteins. (A) Age of patients with low hsCRP <2mg/L (42.5 ± 1.112 years, n = 161) and high hsCRP >2mg/L (43.19 ± 1.339 years, n = 108; p = 0.70) were analyzed. (B) CEC efflux normalized to HDL-C in patients with low (0.1129 ± 0.00463% per ug HDL-C, n = 36; p = 0.70) and high (0.1165 ± 0.007722, n = 32% per ug HDL-C) hsCRP. (C) oxLDL measurements in low (59926 ± 3171, n = 155 mU/L) and high (71125 ± 5723, n = 98; p = 0.09) hsCRP groups. * p<0.05, ** p<0.01, ***p<0.001 and ****p<0.0001.
(TIF)

S3 Fig Flow cytometric analysis of LOX-1 on dendritic cells and inflammatory monocytes. Flow cytometric analysis was performed on PBMCs from SLE patients (n = 17) and healthy individuals (n = 15) for LOX-1 expression. (A) After gating for CD45+ cells and excluding T cells, B cells and granulocytes, LOX-1 expression on inflammatory monocytes HLADR^+^/CD14^+^/CD16^+^ were assessed. (B) After gating for CD45^+^ cells and excluding T cells, B cells and granulocytes and monocytes, HLADR^+^/CD141^+^/CD11c^+^ staining was used to determine LOX-1 expression on mDC1 and HLADR^+^/CD141^-^/CD11c^+^ staining was used to determine LOX-1 expression on mDC2. Spearman correlation (r_s_) between numbers of cells and matched sLOX-1 are also depicted (right panel).
(TIF)

S4 Fig Quantification of CD14^+^ monocytic MDSCs and CD15^+^ LDGs from SLE patients. Flow cytometric quantification of CD3^-^/CD19^-^/CD20^-^/CD56^-^/HLADR^-^/CD33^+^/CD11b^+^ cells separated based on CD14 (monocytic MDSCs) and CD15 (LDGs) expression in SLE patients and healthy donors.
(TIF)

S1 File Raw data file. (XLSX)
