# Supplementary material for: LOX-1: A potential driver of cardiovascular risk in SLE patients
Source: PLoS One. 2020 Mar 17;15(3):e0229184. doi: 10.1371/journal.pone.0229184 (PMC7077835; doi:10.1371/journal.pone.0229184)
Supplement: S1 Table — (DOCX) [file pone.0229184.s001.docx]

**S1 Table. Description of healthy and SLE cohort.**

|  | **Healthy Donors** | **SLE Donors** |  |
| --- | --- | --- | --- |
| Total (n) | 72 | 273 |  |
| Age (years) |  | 42.8 ± 0.85 |  |
| Sex |  | 92 | % Female |
|  |  | 8 | % Male |
| Ethnicity |  | 63 | % Non-Hispanic |
|  |  | 37 | % Hispanic |
| Race |  | 27.47 | % Black |
|  |  | 18.68 | % White |
|  |  | 16.85 | % Asian |
|  |  | 37.00 | % Other |
