# Supplementary material for: LOX-1: A potential driver of cardiovascular risk in SLE patients
Source: PLoS One. 2020 Mar 17;15(3):e0229184. doi: 10.1371/journal.pone.0229184 (PMC7077835; doi:10.1371/journal.pone.0229184)
Supplement: S2 Table — Adjustment for FRS. Unadjusted/adjusted associations between sLOX-1 (dependent variable) and triglycerides, oxLDL, CEC and TACE activity. Adjustment for FRS. Observations that are in ‘bold’ denote significant findings. (DOCX) [file pone.0229184.s002.docx]

**S2 Table. Association between sLOX-1 (dependent variable) and triglycerides, oxLDL, CEC and TACE activity. Adjustment for FRS.**

| **Observation** | **Low LOX-1 (n)** | **Low LOX-1 (Median (IQR))** | **High LOX-1 (n)** | **High LOX-1 (Median (IQR))** | **P value**  **(Group comparison)** | **Spearman Correlation (r_s_)** | **P value (r_s_)** |
| --- | --- | --- | --- | --- | --- | --- | --- |
| **Unadjusted** |  |  |  |  |  |  |  |
| **Triglycerides mg/L** | 163 | 92 (73-137) | 99 | 122 (75-166) | **0.02** | 0.22 | **4.00E-04** |
| **oxLDL (mU/L)** | 163 | 47515  (35967-68392) | 93 | 56385  (42302-85099) | **0.04** | 0.20 | **1.00E-03** |
| **CEC (%efflux/ug HDL-C)** | 36 | 0.12 (0.09-0.13) | 33 | 0.098 (0.07-0.12) | **0.05** | 0.02 | 0.80 |
| **TACE activity (FU/min/ug)** | 170 | 45.71 (32.8-68.4) | 103 | 70.02 (52.8-99.1) | **2.42E-08** | 0.42 | **3.01E-13** |
| **Adjusted** |  |  |  |  |  | **β value** | **P value** |
| **Triglycerides mg/L** | 163 |  |  |  |  | 0.168 | **5.83E-03** |
| **oxLDL (mU/L)** | 163 |  |  |  |  | 0.194 | **1.15E-03** |
| **CEC (%efflux/ug HDL-C)** | 36 |  |  |  |  | -0.0435 | 0.723 |

Unadjusted/adjusted associations between sLOX-1 (dependent variable) and triglycerides, oxLDL, CEC and TACE activity. Adjustment for FRS. Observations that are in ‘bold’ denote significant findings.
