# Supplementary material for: LOX-1: A potential driver of cardiovascular risk in SLE patients
Source: PLoS One. 2020 Mar 17;15(3):e0229184. doi: 10.1371/journal.pone.0229184 (PMC7077835; doi:10.1371/journal.pone.0229184)
Supplement: S3 Table — (DOCX) [file pone.0229184.s003.docx]

**S3 Table. Association between sLOX-1 and SLE medications**

| **Observation** | **Low LOX-1 (n)** | **Low**  **LOX-1 (%)** | **High LOX-1 (n)** | **High**  **LOX-1 (%)** | **P value**  **(Group comparison)** |
| --- | --- | --- | --- | --- | --- |
| **Medication** |  |  |  |  |  |
| Oral Corticosteroids low (<10mg) | 170 | 68.82% | 103 | 66.99% | 0.79 |
| Oral Corticosteroids high (>15mg) | 170 | 15.29% | 103 | 12.62% | 0.6 |
| Hydroxychloroquine | 170 | 72.35% | 103 | 79.61% | 0.2 |
| Azathioprine | 170 | 12.35% | 103 | 9.71% | 0.56 |
| Cyclophosphamide | 170 | 10% | 103 | 6.80% | 0.51 |
| Mycophenolic Acid | 170 | 29.41% | 103 | 32.04% | 0.68 |
| Methotrexate | 170 | 7.06% | 103 | 8.74% | 0.64 |
| Rituximab | 170 | 1.76% | 103 | 2.91% | 0.68 |
| Rapamycin | 170 | 0.59% | 103 | 0% | 1 |
