# Supplementary figures and images for: LOX-1: A potential driver of cardiovascular risk in SLE patients
Source: PLoS One. 2020 Mar 17;15(3):e0229184. doi: 10.1371/journal.pone.0229184 (PMC7077835; doi:10.1371/journal.pone.0229184)

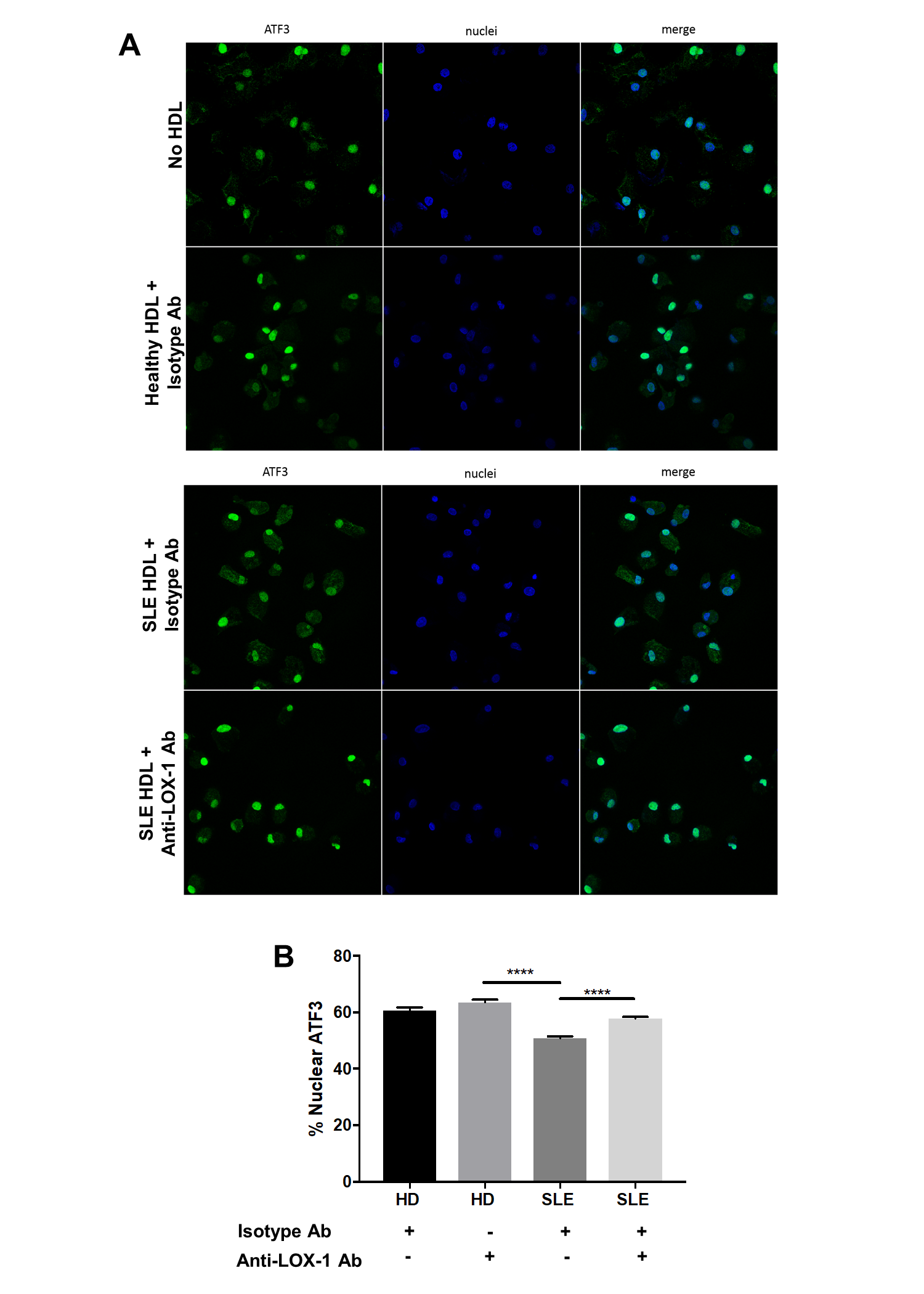

Supplement: S1 Fig — (A) Confocal imaging of primary macrophages exposed to HDL from healthy donors and SLE high sLOX-1 patients. (B) Quantification of nuclear localization of inflammation resolution factor ATF3 in response to exposure to HDL, or blocking with anti-LOX-1 Ab prior to exposure to HDL. (TIF) [file pone.0229184.s004.tif]

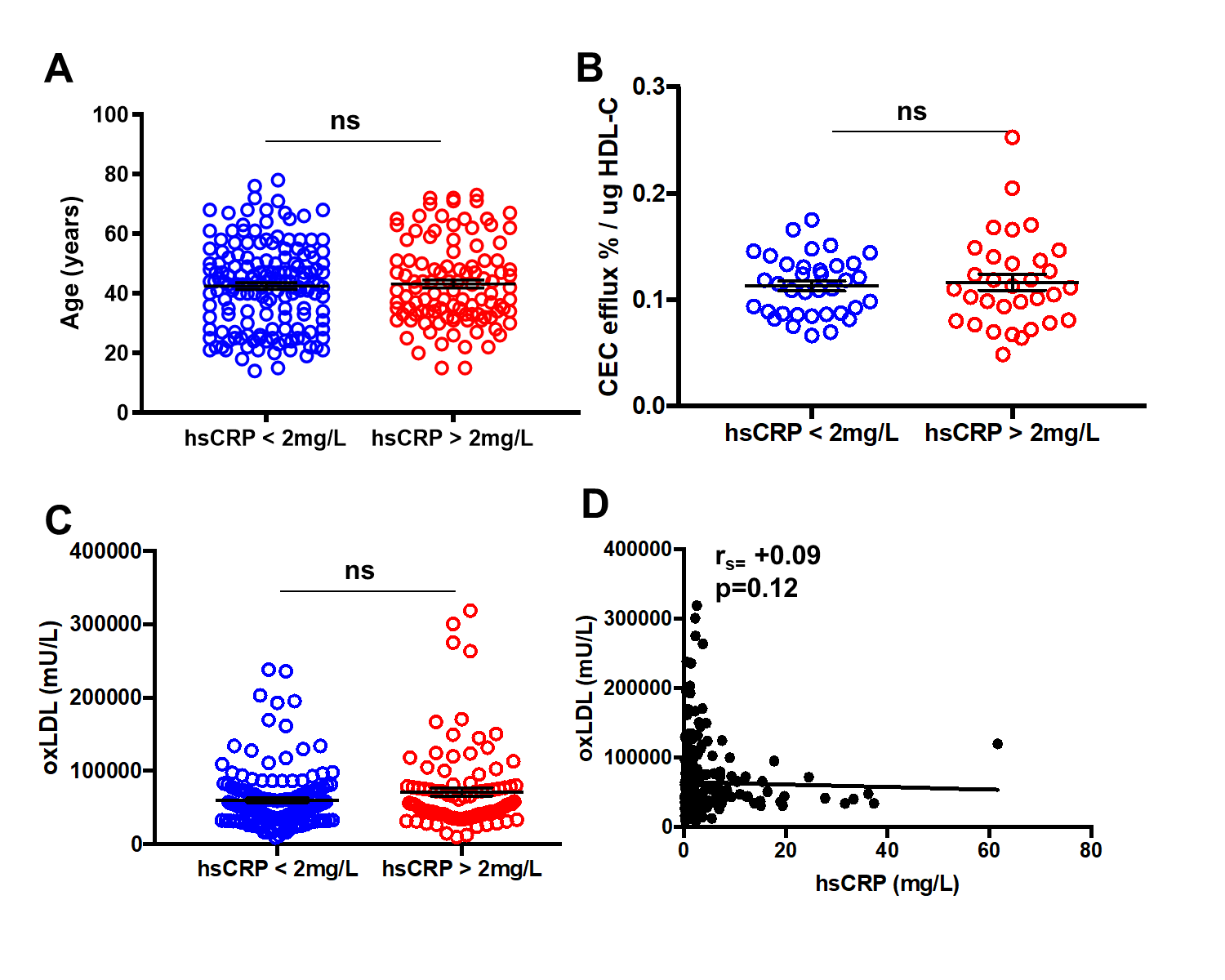

Supplement: S2 Fig — (A) Age of patients with low hsCRP <2mg/L (42.5 ± 1.112 years, n = 161) and high hsCRP >2mg/L (43.19 ± 1.339 years, n = 108; p = 0.70) were analyzed. (B) CEC efflux normalized to HDL-C in patients with low (0.1129 ± 0.00463% per ug HDL-C, n = 36; p = 0.70) and high (0.1165 ± 0.007722, n = 32% per ug HDL-C) hsCRP. (C) oxLDL measurements in low (59926 ± 3171, n = 155 mU/L) and high (71125 ± 5723, n = 98; p = 0.09) hsCRP groups. * p<0.05, ** p<0.01, ***p<0.001 and ****p<0.0001. (TIF) [file pone.0229184.s005.tif]

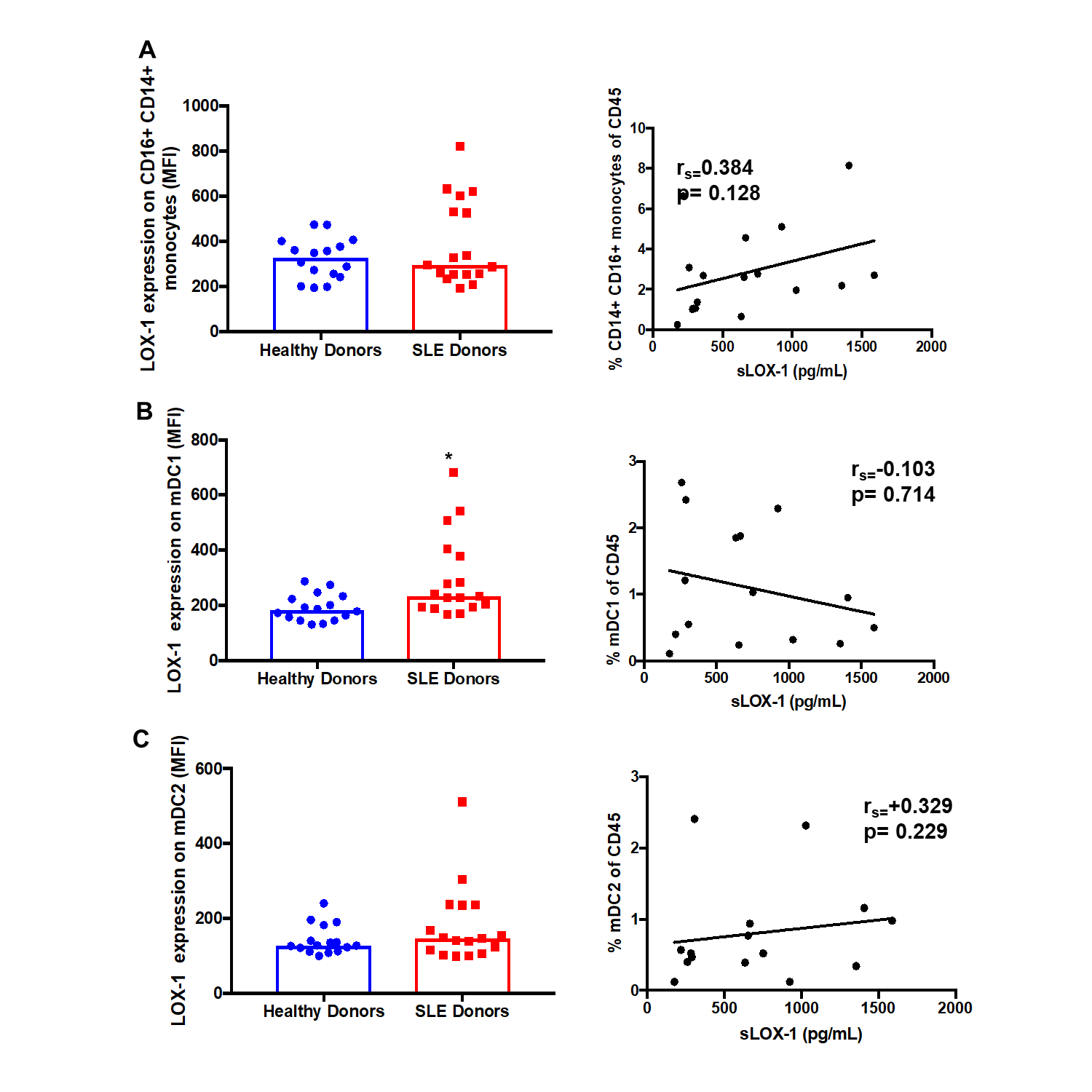

Supplement: S3 Fig — Flow cytometric analysis was performed on PBMCs from SLE patients (n = 17) and healthy individuals (n = 15) for LOX-1 expression. (A) After gating for CD45+ cells and excluding T cells, B cells and granulocytes, LOX-1 expression on inflammatory monocytes HLADR+/CD14+/CD16+ were assessed. (B) After gating for CD45+ cells and excluding T cells, B cells and granulocytes and monocytes, HLADR+/CD141+/CD11c+ staining was used to determine LOX-1 expression on mDC1 and HLADR+/CD141-/CD11c+ staining was used to determine LOX-1 expression on mDC2. Spearman correlation (rs) between numbers of cells and matched sLOX-1 are also depicted (right panel). (TIF) [file pone.0229184.s006.tif]

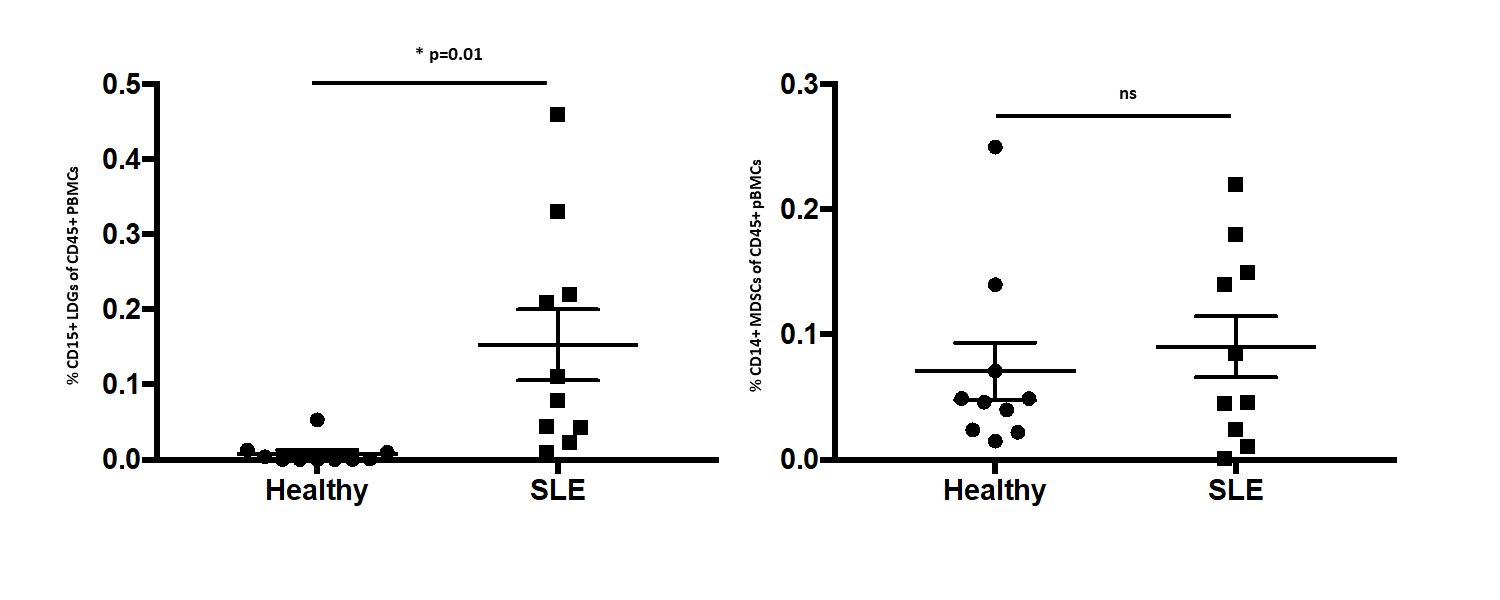

Supplement: S4 Fig — Flow cytometric quantification of CD3-/CD19-/CD20-/CD56-/HLADR-/CD33+/CD11b+ cells separated based on CD14 (monocytic MDSCs) and CD15 (LDGs) expression in SLE patients and healthy donors. (TIF) [file pone.0229184.s007.tif]
